# About inflammation and infection

**DOI:** 10.1186/2191-219X-3-8

**Published:** 2013-02-01

**Authors:** Alberto Signore

**Affiliations:** 1 Nuclear Medicine Unit, Department of Medical-Surgical Sciences and of Translational Medicine, Sapienza University, Rome, Italy

## Abstract

This letter points out the correct definition of inflammation and infection that is important for differential diagnosis. The most common nuclear medicine techniques and interpretation criteria to differentiate inflammation between infection are also briefly mentioned.

## Correspondence

Dear Editor,

The review recently published by Autio et al. entitled ‘Nuclear imaging of inflammation: homing-associated molecules as targets’ [1] contains some statement that may be misleading and requires some clarification.

In particular, I think it is important to correctly define the terms ‘inflammation’ and ‘infection.’ Just by reading the definition of inflammation in the Wikipedia, we learn that ‘inflammation is part of the complex biological response of vascular tissues to harmful stimuli, such as pathogens, damaged cells, or irritants. The classical signs of acute inflammation are pain (*dolor*), heat (*calor*), redness (*rubor*), swelling (*tumor*), and loss of function (*functio laesa*). Inflammation is a protective attempt by the organism to remove the injurious stimuli and to initiate the healing process. Inflammation is not a synonym for infection, even in cases where inflammation is caused by infection. Although infection is caused by a microorganism, inflammation is one of the responses of the organism to the pathogen. However, inflammation is a stereotyped response, and therefore it is considered as a mechanism of innate immunity, as compared to adaptive immunity, which is specific for each pathogen’ [2].

Similarly, we can find a definition for infection as ‘the invasion of a host organism's bodily tissues by disease-causing organisms, their multiplication, and the reaction of host tissues to these organisms and the toxins they produce. Infections are caused by microorganisms such as viruses, prions, bacteria, and viroids, and larger organisms like parasites and fungi. Hosts can fight infections using their immune system. Mammalian hosts react to infections with an innate response, often involving inflammation, followed by an adaptive response’. Therefore, we usually always have an inflammation associated with an infection, but not always we have an infection if there is an inflammation [3].

This is not just an exercise of semantics, but it is very relevant, particularly for the nuclear medicine point of view. Indeed, nuclear medicine techniques aim at differentiating ‘sterile inflammation’ from infection and the two terms cannot be used as synonyms.

It emerges, as a consequence of what we defined above, that a good radiopharmaceutical for imaging infection should not image inflammation. This is not always easy, and a certain amount of radiopharmaceutical accumulation in sites of sterile inflammation can often be noticed when seeking for infection. In case of radiolabeled white blood cells (WBC) and radiolabeled anti-granulocyte monoclonal antibodies, the specificity for infection can be improved by optimizing the image acquisition and interpretation protocols. These protocols are currently being standardized by the EANM Committee on infection/inflammation imaging, but most users of WBC already know and successfully apply these criteria. In short, images should be acquired at three time points (30 min to 1 h, also called ‘early image’; 3 to 4 h, also called ‘delayed image’; and 20 to 24 h post-injection, also called ‘late image’) in a time-corrected manner for isotope decay. Then, images are displayed with the same intensity scale, and any focal increase of activity or size with time should be considered an infection, whereas an accumulation at 3 to 4 h with a decrease at 20 to 24 h is a sign of a sterile inflammation. As mentioned, it is important that in papers and reviews, we learn to use the correct terminology in order not to create confusion to the readers.

The sentence reported in the Abstract by Autio et al. ‘the golden standard in nuclear medicine imaging of inflammation is the use of autologous radiolabeled leukocytes’ is therefore misleading, being WBC the gold standard technique for imaging infection.

Furthermore, in the Introduction, it is reported that ‘non-invasive imaging of inflammation could be a highly valuable tool as it could help diagnosing many inflammatory conditions, such as osteomyelitis, rheumatoid arthritis, sarcoidosis, inflammatory bowel disease, and fever of unknown origin.’ These diseases cannot be pooled together. We should clarify that the aim of nuclear medicine in osteomyelitis is to image infection. In sarcoidosis and rheumatoid arthritis, we aim at imaging inflammation, and therefore, many ‘granulocyte-based approaches’ are useless and FDG and anti-TNFa MoAb seem much better agents by targeting monocytes and other inflammatory cells/components [4]. In IBD and FUO, the situation is much more complicated as we might need to image either the inflammatory events or the sites of pathological granulocyte accumulation (abscesses, fistulae, inflammatory stenosis, etc.).

In all cases, it is extremely important to keep a distinction between inflammation and infection, to use the appropriate terminology, and to keep in mind that we have radiopharmaceuticals designed specifically for infection imaging and others that are more appropriate for sterile inflammation imaging (such as sarcoidosis, vasculitis, rheumatoid arthritis, atherosclerosis, autoimmune diseases, degenerative diseases, etc.). This is not clearly emerging from the review of Autio et al. and may lead to some confusion.

## Competing interests

The author declares that he has no competing interests.
